# Treatment satisfaction and efficacy of the rapid release formulation of sumatriptan 100 mg tablets utilising an early intervention paradigm in patients previously unsatisfied with sumatriptan

**DOI:** 10.1111/j.1742-1241.2008.01935.x

**Published:** 2008-12

**Authors:** L C Newman, R K Cady, S Landy, P O’Carroll, W J Kwong, S P Burch, A C Nelsen, S A McDonald

**Affiliations:** 1 The Headache Institute, Roosevelt Hospital CenterNew YorkAlbert Einstein College of Medicine BronxNY, USA; 2 Primary Care NetworkSpringfield, MO, USA; 3 Wesley Headache Clinic, University of Tennessee School of MedicineMemphis, TN, USA; 4 400 Newport Center Dr.Newport Beach, CA, USA; 5 Department of Clinical and Administrative Pharmacy, University of GeorgiaAthens, GA, USA; 6 Health Management Innovations, GlaxoSmithKline, Research Triangle ParkNC, USA; 7 Pharmacogenetics, GlaxoSmithKline, Research Triangle ParkNC, USA; 8 MDC, Neurosciences, US Clinical, Migraine, GlaxoSmithKline, Research Triangle ParkNC, USA

## Abstract

**Aims:**

To evaluate treatment satisfaction, efficacy and functional ability of the rapid release formulation of sumatriptan 100 mg tablets (sumatriptan RT 100 mg) in an early intervention paradigm in patients who were dissatisfied with low-dose sumatriptan and not completely satisfied with their current migraine regimen.

**Methods:**

Experienced migraineurs who reported a mild migraine pain phase, dissatisfaction with the previous sumatriptan treatment and some dissatisfaction with their current treatment regimen had no experience with sumatriptan at the 100 mg dose were enrolled in an open-label, single group study. Subjects were instructed to treat four migraine attacks within 30 min of the onset of mild pain. Treatment satisfaction was measured with the Patient Perception of Migraine Questionnaire Revised version (PPMQ-R) questionnaire.

**Results:**

More than half of the subjects were either very satisfied or satisfied with the efficacy of early intervention sumatriptan RT 100 mg after each attack and at the follow-up study visit. The mean total PPMQ-R score was 75.2 out of 100. Between 63% and 73% of subjects were pain-free within 4 h of dosing. Between 79% and 90% of subjects reported an ability to function normally within 4 h of taking the study medication.

**Conclusion:**

Subjects who were previously unsatisfied with lower doses of sumatriptan and less than very satisfied with their current treatment regimen were more likely to be satisfied or very satisfied with sumatriptan RT 100 mg in an early intervention paradigm. Results were consistent across four migraine attacks and at a follow-up visit. The treatment satisfaction results corresponded with positive results on efficacy measures and a functional status measure.

**What’s known**

Sumatriptan RT 100 mg tablets effectively treat migraine headaches in an early treatment paradigm.

**What’s new**

Patients who were previously unsatisfied with lower doses of conventionally formulated and other formulations of sumatriptan therapy are more likely to report that they are satisfied with sumatriptan RT 100 mg treatment when used in an early intervention paradigm.

## Introduction

Migraine is a chronic condition characterised by episodic attacks of disabling headaches that are generally pulsatile in nature and often associated with gastrointestinal disturbances, photophobia, phonophobia and neurological disruptions in cognition and mood (1). In some migraineurs, focal neurological disruptions, called auras, occur prior to the onset of headache. In the United States, approximately 18% of women suffer from at least one migraine headache per year compared with 6% of men (2). The prevalence of migraine peaks during the most productive adult years (25–55 years of age) creates a significant social and economic burden (3,4). One study found that the healthcare costs for a family with a migraineur were 70% higher than for the matched families without a migraine sufferer (5).

For patients who seek migraine treatment through their healthcare providers, a member of the triptan drug class may be prescribed for the acute treatment of migraine attacks (6). The efficacy of triptans for severe migraines was established in clinical trials first with sumatriptan (7–10). The evaluation of triptans for the treatment of moderate-to-severe migraines was required by regulatory authorities, and the primary end-point in these early studies was the reduction in moderate or severe pain to mild or no pain (11). In this treatment paradigm, subjects who experienced mild headaches as a prelude to moderate-to-severe headaches were instructed to delay treatment until their headache pain was moderate or severe (12). The results of the recently published clinical evidence and pathophysiological studies suggest that the drugs in the triptan class provide greater efficacy when administered during the mild headache phase of migraine, an early intervention paradigm, rather than delaying treatment and sustaining the disability of a moderate-to-severe headache (13–20). Modelling studies suggest that early intervention is also more cost-effective than waiting for moderate or severe pain to develop because early intervention more effectively controls pain and reduces the need for rescue medication (21,22). Controlled clinical trials with sumatriptan have shown that early intervention improves pain-free efficacy and reduces migraine pain and symptom duration (15,17,19). The International Headache Society now recommends that efficacy studies for migraine treatments include studies of medications in an early intervention paradigm (11).

In an early intervention paradigm, the optimal dose and rapid delivery of the medication may improve effectiveness and treatment satisfaction by preventing progression in the migraine cycle. Multiple studies of conventional sumatriptan administered in an early intervention paradigm have demonstrated that the 100 mg dose is more efficacious than lower doses at providing pain-free response in a clinical trial setting (15,18,19,23). Recently, oral sumatriptan was reformulated with RT technology to disperse rapidly and disintegrate in the stomach to improve absorption. Gastric scintigraphic evaluation of the reformulated and conventional tablets confirms that the reformulated tablet disperses, disintegrates and is absorbed faster in migraineurs (24). Randomised controlled trials confirm that the reformulated tablets are efficacious (25,26).

The objectives of this study were to evaluate treatment satisfaction, efficacy and functional ability associated with the rapid release formulation of sumatriptan 100 mg tablets (sumatriptan RT 100 mg) in an early intervention paradigm (within 30 min of onset of pain and while still mild) among patients who were dissatisfied with the previous low-dose sumatriptan treatment and who were not completely satisfied with their current migraine treatment regimen. Patient reported outcomes such as treatment satisfaction are increasingly considered end-points that encompass the overall treatment experience (27). The Federal Drug Administration (FDA) is encouraging the medical research community to measure patient reported outcomes and has released a draft guidance document with the goal of describing how the FDA will evaluate effectiveness data collected using patient reported outcomes measures in clinical trials (28). By providing an overview of the patient’s perspective of treatment successes or shortcomings, treatment satisfaction results provide clinically meaningful information to help inform treatment decisions.

## Methods

### Study population

The study population included female and male migraineurs aged 18–65 years with a diagnosis of migraine, with or without aura (2004 IHS Criteria ICHD-II diagnosis 1.2.1 and 1.1) (1), at least a 1-year history of migraine headaches and an average of 2–6 migraine attacks per month in the 2 months prior to screening. Subjects were required to report that they typically experience a mild pain phase preceding a moderate to severe pain phase of migraine, that they could distinguish mild-onset migraine headaches from other types of headache, that they had been dissatisfied with previous sumatriptan treatment in any formulation (e.g. tablet, subcutaneous injection or nasal spray) at a dose < 100 mg and that they were less than very satisfied with their current acute treatment regimen. At the time of enrolment, subject satisfaction with their current treatment regimen was measured by a single-item question with a 7-point response scale. Subjects who rated their satisfaction with current treatment as ‘very satisfied’ were excluded from the study.

Subjects who were using sumatriptan as part of their current migraine treatment regimen were excluded because discontinuation of the previous sumatriptan therapy was viewed as a sign of dissatisfaction with treatment. To measure patient satisfaction with a new therapy at a higher dose, patients who had used the 100 mg dose of sumatriptan prior to the study start were also excluded.

### Study design

Treatment satisfaction, treatment efficacy and functional status were measured in a multi-centre, open-label, single group, prospective study. Subjects were required to attend two study visits: one at study entry (screening) and one at study exit (follow up). Follow-up visits were conducted within 2 weeks of the last treated attack or at 8 weeks after the screening visit.

### Procedures

At the screening visit, subjects were provided with four tablets of sumatriptan RT 100 mg for the initial treatment of mild migraine headaches and five tablets of sumatriptan RT 100 mg for rescue use during the 2–24 h after the initial dose of study medication, if needed. Other rescue medications (excluding ergotamine, ergot-type medications and other triptans) were permitted when taken at least 2 h after the initial dose of study medication and documented in the patient diary. Subjects were not required to meet a minimum level of pain severity as a prerequisite for taking rescue medications. Ergotamine or ergot-type medications (e.g. dihydroergotamine and methysergide) and other triptans were not allowed during the 24 h before or after administration of the study medication.

Subjects were instructed to treat four consecutive migraine attacks within 30 min of the onset of mild pain and to refrain from using study medication to treat attacks with moderate or severe pain at onset. Subjects were allowed to continue migraine prophylactic medications provided the medication had been included in their previous regimen for at least 1 month prior to screening.

### Measures

Subjects were instructed to complete a patient diary entry for each attack treated with study medication. The patient diary required subjects to record the date and time migraine pain started, the date and time of the first dose of study medication, the pain severity level, the presence or absence of symptoms and the level of functional impairment. The treatment satisfaction questionnaire was included in the patient diary. The diary also captured the date, time and the type of rescue medication; the pain severity level at the time of rescue; and all medications, excluding study medications and daily medications, taken 24 h before and after the study medication doses.

In the patient diary, subjects recorded their pain severity level, associated symptoms and functional ability at the time of dosing and 30 min, 1, 2, 4 and 24 h after dosing. Pain severity level was measured on a 4-point scale (‘none’, ‘mild’, ‘moderate’ or ‘severe’). Subjects reported the presence or absence of nausea, vomiting, photophobia and phonophobia. Subjects recorded their ability to function on a 5-point scale (‘not impaired’, ‘mildly impaired’, ‘moderately impaired’, ‘severely impaired’ or ‘required bed rest’).

Treatment satisfaction with sumatriptan RT 100 mg was assessed using the Patient Perception of Migraine Questionnaire Revised version (PPMQ-R). Subjects were instructed to complete the PPMQ-R at 24 h after taking the first dose of study medication for each treated attack. Subjects also completed the PPMQ-R at the follow-up visit.

The PPMQ-R is a validated patient satisfaction instrument containing 32 items that contribute to four scales: Tolerability (10 items), Efficacy (11 items), Functionality (four items) and Ease of Use (two items). The items contributing to the Efficacy, Functionality and Ease of Use scales were scored on a scale ranging from 1 (very dissatisfied) to 7 (very satisfied). The items contributing to the Tolerability scale were scored on a scale ranging from 1 (extremely bothersome) to 5 (not at all bothersome). A total score was calculated based on the average of scores for Efficacy, Functionality and Ease of Use (29,30).

In addition to the items contributing to the Efficacy, Functionality, Ease-of-Use and Tolerability scales, the PPMQ-R contains three global satisfaction items – one each measuring overall satisfaction, satisfaction with medication effectiveness and satisfaction with side effects. The global satisfaction items were scored on a scale ranging from 1 (very dissatisfied) to 7 (very satisfied) (29,30).

Two PPMQ-R items designed to measure satisfaction with drug cost were excluded from the study questionnaire because study medication was provided as part of the clinical trial. The PPMQ-R has demonstrated reliability and validity in measuring patient satisfaction with acute migraine treatment (29,30).

At the follow-up visit, subjects returned unused study drug and completed diaries. Subjects also completed follow-up questions and the PPMQ-R. For the PPMQ-R, subjects were instructed to consider their experience with the study drug across all attacks treated during the study.

### Analysis

All subjects who took at least one dose of sumatriptan RT 100 mg were included in the safety population. The intention to treat (ITT) population included subjects who treated at least one attack according to the treatment instructions (i.e. migraines were treated within 30 min of the onset of the migraine headache pain and during a mild pain phase). Data from attacks in which subjects did not comply with the protocol with respect to treatment instructions were not included in the analyses. As a result, summaries on a per-attack basis will have different denominators based on the number of observations from subjects who treated the specific attack according to the treatment instructions.

For the PPMQ-R, mean scores were calculated from the item scores for each of the four scales (Efficacy, Functionality, Ease of Use and Tolerability). Each scale score ranges from 0 to 100, with higher scores indicating greater satisfaction or better tolerability. A total score, a composite of the scale scores for Efficacy, Functionality and Ease of Use, was also computed. Previous research suggests that a 5-point difference in the Efficacy, Functionality and Ease-of-Use scale scores and the Total score is clinically meaningful (26,27). Global satisfaction items (effectiveness, side effects and overall) were summarised as percentage of subjects by response option.

Descriptive statistics were calculated for efficacy and functional ability end-points. The percent of patients who were pain-free at each time point was calculated. The percent of patients reporting the presence of nausea, photophobia and phonophobia was calculated at dosing and each time point. The percent of patients reporting normal functioning at dosing and 30 min, 1, 2, 4 and 24 h after dosing was calculated.

## Results

### Subject characteristics at baseline and demographic data

A total of 140 subjects from six sites in the United States were enrolled and 134 (96%) returned their patient diaries. The safety population consisted of 120 subjects who were randomised and treated. The ITT population included 105 subjects who treated at least one migraine according to the study instructions. In the safety population, 9% withdrew prematurely. Three were lost to follow up, one because of an adverse event, one withdrew consent, one for an unspecified protocol violation and four for unknown or other reasons.

A majority of subjects in the ITT population were women (88%) and Caucasian (88%). The average age of study subjects was 41 years (Table 1). The mean number of migraines per month at baseline was 3.9, and more than 99% of subjects reported that their migraines typically lasted more than 4 h. Of the subjects in the ITT population, 43% reported that they typically have migraines lasting more than 24 h. Subjects reported a median of 24 years since onset of migraine disease, and 80% reported experiencing migraines for more than 10 years. Severe migraines were reported by 75% of the subjects. More than half of all subjects (68%) were diagnosed with migraine without aura compared with 8% who were diagnosed with migraine with aura, and 23% who were diagnosed with migraine with and without aura.

**Table 1:** Demographics and migraine type for the ITT population

	ITT population (*n*= 105)*
Mean age (SD)	41.2 years (11.2 years)
**Gender, *n/N* (%)**	
Female	90/102 (88%)
Male	12/102 (12%)
**Race, *n/N* (%)**	
Caucasian	90/102 (88%)
Asian	4/102 (4%)
African American	1/102 (<1%)
Hispanic	3/102 (3%)
Other	4/102 (4%)
**Previous form of sumatriptan therapy, *n/N* (%)**	
Tablet, 25 mg	39/105 (37%)
Tablet, 50 mg	68/105 (65%)
Subcutaneous injection	39/105 (37%)
Nasal spray	40/105 (38%)
**Migraine type†, *n/N* (%)**	
Without aura	71/105 (68%)
With aura	8/105 (8%)
With and without aura	24/105 (23%)
Unknown	2/105 (2%)

* Demographic information was missing for three subjects in the ITT population. †Migraines type was defined by the ICHD-2 classification from the IHS (1). ITT, intention to treat.

Of the subjects who had discontinued previous sumatriptan therapy at a dose lower than 100 mg, more patients had taken sumatriptan in the 50 mg tablet form (65%) than any other form (Table 1). The most commonly reported reason for discontinuation was lack of efficacy.

In the 3 months prior to study enrolment, 93% of subjects reported using any pharmacological treatment. Pharmacological treatments included other triptans (55%), over-the-counter analgesics (46%), narcotics (22%) and prescription NSAIDS (19%). As per the protocol, no subject reported that he/she was very satisfied with the current treatment regimen. Three percent (3 of 101) were satisfied with their current migraine mediations and 37% were somewhat satisfied with their current migraine medications. Five percent of subjects were neither satisfied nor dissatisfied with their current treatment. A total of 35% of subjects were somewhat dissatisfied, 13% were dissatisfied and 8% were very dissatisfied with their current migraine treatment regimen.

### Satisfaction results

#### Global satisfaction items from the PPMQ-R

**Figure 1:**
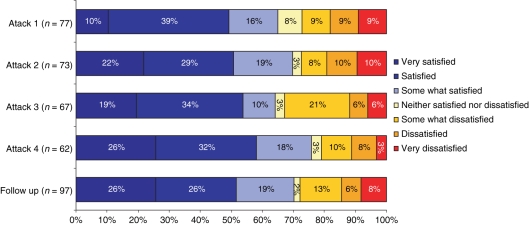
Percent of subjects by reported level of overall satisfaction with study treatment by attack and at follow-up visit as measured by the PPMQ-R

Based on the results from the PPMQ-R overall satisfaction question measured at the follow-up visit, 26% of subjects who were not very satisfied with their previous treatment regimen were very satisfied with sumatriptan RT 100 mg in an early intervention paradigm (Figure 1). More than 50% of subjects were either very satisfied or satisfied with the study drug for attacks 2 through 4 and at follow up. Thirty-eight of 77 subjects (49%) were satisfied or very satisfied after treating the first attack.

**Figure 2:**
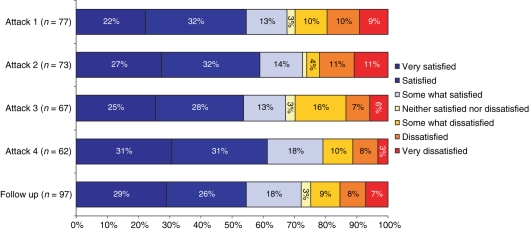
Percent of subjects by reported level of satisfaction with the efficacy of study treatment by attack and at follow-up visit as measured by the PPMQ-R

**Figure 3:**
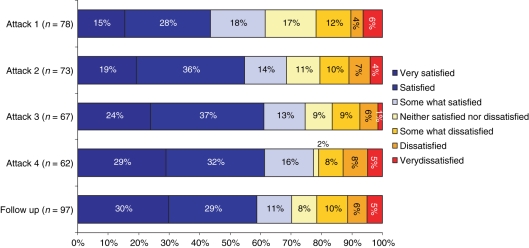
Percent of subjects by reported level of satisfaction with the side effects of study treatment by attack and at follow-up visit as measured by the PPMQ-R

More than half of all subjects were either very satisfied or satisfied with the efficacy of early intervention sumatriptan RT 100 mg after each attack and at the follow-up study visit (Figure 2). With the exception of the first attack, more than half of all subjects were very satisfied or satisfied with side effects associated with the study drug (Figure 3). Less than 15% of subjects were dissatisfied or very dissatisfied with the side effect profile of sumatriptan RT 100 mg following all attacks and at the follow-up visit.

#### PPMQ-R Scale scores and total score

**Figure 4:**
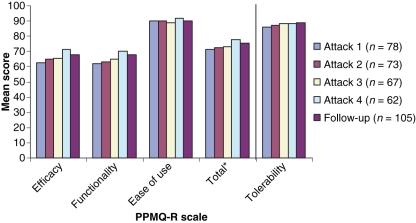
Mean PPMQ-R Scores by scale for each attack and at follow up. *Total score is a composite of Efficacy, Functionality and Ease of Use scales. Higher scores indicate a higher level of treatment satisfaction and better tolerability

The mean PPMQ-R scores are presented in Figure 4 for each of the fours attacks and at the follow-up visit. For the Efficacy scale, the mean score was > 60 for all four attacks and at the follow-up visit. Mean scores for Functionality ranged from 61.8 for attack 1 to 70.2 for attack 4. At the follow-up visit, the mean score for Functionality was 67.9. The mean Ease of Use scores ranged from 88.8 for attack 3 to 91.9 for attack 4. The mean score for Ease of Use was 90.1 at the follow-up visit. For the Tolerability scale of the PPMQ-R, a higher score indicates better tolerability and the mean score at final visit was 88.6. The total score is calculated by the average of Efficacy, Functionality and Ease of Use scale scores. For attack 1, the mean total score was 71.4. For attacks 2, 3 and 4, the mean scores were 72.7, 73.2 and 77.9 respectively. The mean score at follow up was 75.2.

#### Efficacy and functional impairment results

**Figure 5:**
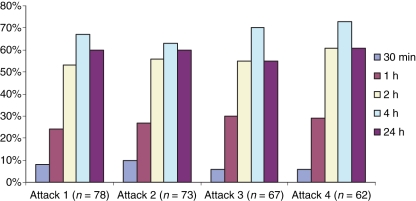
Percentage of subjects who were pain-free as a function of time for each attack

Between 53% (attack 1) and 61% (attack 4) of subjects reported being pain-free within 2 h of taking the first dose of study medication (Figure 5). Within 4 h of taking the first dose of study medication, between 63% (attack 2) and 73% (attack 4) of subjects were pain-free. Between 35% (attack 1) and 45% (attack 3) of subjects had taken either a second dose of study drug or another approved rescue medication between 2 and 24 h after the initial dose (Table 2) The percentage of subjects taking migraine prophylaxis ranged from 23% (attack 4) to 28% (attack 1) (Table 2).

**Table 2:** Number and percentage of patient taking rescue medications and migraine prophylaxis by attack

	Attack 1 (*n*= 78)	Attack 2 (*n*= 73)	Attack 3 (*n*= 67)	Attack 4 (*n*= 62)
Rescued: *n* (%)	27 (35%)	26 (36%)	30 (45%)	22 (35%)
Mean (SD) hours to rescue	8.8 (7.5)	8.6 (8.2)	9.0 (7.4)	10.0 (8.3)
**Pain severity at rescue: *n* (%)**				
Mild	10 (37%)	14 (54%)	16 (53%)	13 (59%)
Moderate	15 (56%)	7 (27%)	11 (37%)	5 (23%)
Severe	2 (7%)	5 (19%)	3 (10%)	4 (22%)
Migraine prophylaxis	22 (28%)	20 (27%)	17 (25%)	14 (23%)

Rescue medications include those the subject said were for migraine pain or a second dose of study drug taken 0–24 h after initial dose of study drug.

When compared with symptoms at the time of dosing, reports of nausea, photophobia and phonophobia were lower at 2, 4 and 24 h postdose for all four attacks (Table 3). Across all four attacks, only three reports of vomiting were reported.

**Table 3:** Summary of the incidence of symptoms associated with migraine by attack

	Attack 1	Attack 2	Attack 3	Attack 4
**Symptoms *n/N* (%)**				
**Nausea**				
Present at dosing	21/76 (28%)	25/73 (34%)	18/67 (27%)	23/62 (37%)
Present 2 h after dosing	19/78 (24%)	15/73 (21%)	15/67 (22%)	14/62 (23%)
Present 4 h after dosing	11/78 (14%)	9/73 (12%)	6/67 (9%)	5/62 (8%)
Present 24 h after dosing	4/78 (5%)	3/73 (4%)	5/67 (7%)	1/62 (2%)
**Photophobia *n/N* (%)**				
Present at dosing	46/78 (59%)	47/73 (64%)	47/67 (70%)	45/62 (73%)
Present 2 h after dosing	24/78 (31%)	23/73 (32%)	23/67 (34%)	20/62 (32%)
Present 4 h after dosing	15/78 (19%)	15/73 (21%)	15/67 (22%)	9/62 (15%)
Present 24 h after dosing	10/78 (13%)	8/73 (11%)	10/67 (15%)	3/62 (5%)
**Phonophobia *n/N* (%)**				
Present at dosing	44/77 (57%)	43/73 (59%)	40/67 (60%)	41/62 (66%)
Present 2 h after dosing	20/77 (26%)	23/73 (32%)	22/67 (33%)	20/62 (32%)
Present 4 h after dosing	14/77 (18%)	17/73 (23%)	11/67 (16%)	10/62 (16%)
Present 24 h after dosing	12/77 (16%)	7/73 (10%)	9/67 (13%)	4/62 (6%)

**Figure 6:**
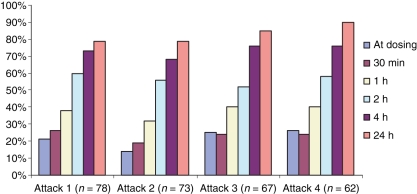
Percentage of subjects who reported normal functional ability at dosing and at specified intervals postdose for each attack

Between 79% (attacks 1 and 2) and 90% (attack 4) of subjects reported an ability to function normally within 24 h of taking the study medication (Figure 6). Following the first attack, 24 subjects (31%) reported that their pain-free status was sustained. Sustained pain-free status was obtained by 20 subjects (27%) for attack 2, 21 subjects (31%) for attack 3 and 23 subjects (37%) for attack 4.

Of the subjects in the ITT population, 65% of subjects in the ITT population reported that they would use this medication again. A total of 85% of subjects reported that the medication was very easy or easy to use, and 94% would treat future migraine attacks within 30 min of onset.

## Discussion

The results of this study suggest that experienced migraineurs who were previously unsatisfied with lower doses of sumatriptan in any form (i.e. tablets, nasal spray or injection) and less than very satisfied with their current treatment regimen are more likely to be satisfied or very satisfied with sumatriptan RT 100 mg in an early intervention paradigm. In this study, the mean scale scores for treatment satisfaction when measured by a validated treatment satisfaction questionnaire indicate that subjects tended to be satisfied with the Efficacy, Ease Of Use, Functionality and side effects of sumatriptan RT 100 mg when treating migraine in an early intervention paradigm. The treatment satisfaction results corresponded with positive results on efficacy measures and a functional status measure.

The percentage of patients who were very satisfied with treatment following the first attack was lower than the percentage at attacks 2, 3, 4, and at the follow-up visit. With any new treatment or intervention, experience impacts the outcome. Global satisfaction may have been slightly lower after the first attack because it was the subjects’ first experience using this intervention (EI paradigm and sumatriptan RT 100 mg) after having reported dissatisfaction with sumatriptan. That is, because these subjects were previously dissatisfied with sumatriptan, they may have had lower expectations at the beginning of the study.

Additionally, the percent of patients who were satisfied and very satisfied with side effects is higher for attacks 3 and 4 than for attacks 1 and 2. While it is possible that patients who remained in the study had fewer or less severe side effects compared with those who dropped out, patients may have become more familiar with the drug and its side effects over the course of the study. In our experience, it is not uncommon for subjects to report fewer adverse events (AEs) over time in long-term studies.

Treatment satisfaction, defined as the subject’s evaluation of important attributes associated with the process and outcomes of treatment, is becoming a more common end-point in clinical trials (27). In an international survey of patients with a migraine diagnosis, only 36% reported that they were ‘very satisfied’ with their current migraine treatment and only 27% reported that their current migraine therapy consistently managed every migraine attack (31). Physicians who treat patients with migraine should be aware that their patients may not be fully satisfied with their treatment regimen and should initiate discussions about treatment options. The results of this open label study suggest that experienced migraineurs might benefit from modifications to their treatment regimen including medication, dose, formulation and timing of treatment administration. Further, the consistency of treatment efficacy over repeated attacks of migraine observed in this study may serve to increase patient confidence and add further to their satisfaction.

Other studies have demonstrated that treatment satisfaction for migraineurs is closely linked with clinical efficacy and that patient preferences are impacted by previous treatment experience (32–36). This study found that the positive results on patient satisfaction measures were consistent with the positive results on efficacy measures and functional status measures. These findings might be attributed to the higher dose of sumatriptan, the fast disintegrating, rapid-release formulation, the early intervention paradigm or a combination of all three.

Multiple studies of conventional sumatriptan administered in an early intervention paradigm have demonstrated that the 100 mg dose is more efficacious than lower doses in a clinical trial setting (15,18,19,23). These studies have also shown that both the 50 and 100 mg doses of conventional sumatriptan were well-tolerated, and no new or unusual side effects were identified at the 100 mg dose (15,18,19,23). In a pooled-analysis of six studies of the early intervention paradigm comparing 50 mg of conventional sumatriptan, 100 mg of conventional sumatriptan and placebo, the results demonstrated that both doses were well-tolerated, and that the 100 mg dose was significantly more efficacious (18). A study of the safety and efficacy of sumatriptan RT at the 50 and 100 mg doses also found that the 100 mg dose of sumatriptan RT was superior to the 50 mg dose on multiple efficacy measures and uncovered no new or unusual side effects at either dose (26). The results of this open-label study support the findings from randomised controlled trials that have demonstrated that sumatriptan RT 100 mg is effective in an early treatment paradigm (25,26).

Improvement in functional status is an important end-point for migraineurs because the majority of the costs associated with migraine results from missed time and productivity loss rather than from direct healthcare costs (37). A productivity study of sumatriptan 100 mg in an early intervention paradigm found the degree of reduction in productivity loss was inversely related to the severity of pain at initial dosing of sumatriptan (38). Improvements in functional status during migraine attacks may help reduce the burden of migraine to patients and to society. Subjects in this study reported improved mean functional status at each time point after the initial dose of study medication.

The results of this study should be interpreted in the context of the limitations. Although it is desirable to evaluate treatment outcomes in a real-world environment, certain biases are more likely to be present in an open-label, uncontrolled study compared with a randomised, controlled study. To reduce the impact of selection bias, we restricted the study population to experienced migraineurs who reported dissatisfaction with the study drug in the previous treatment settings and less than complete satisfaction with their current medication regimen. Although these subjects were less likely to represent migraineurs with a mild condition, their experience with the condition and treatment provided a specific perspective on treatment satisfaction. Lost to follow up can be a significant source of selection bias. In this study, the drop out rate was < 10%, which suggests that the study medication and treatment regimen were well tolerated.

A potential source of recall bias was that subjects were required to remember their previous experiences with sumatriptan to be eligible for the study. The impact of this was minimised by providing potential subjects with only two options: satisfied or dissatisfied. To reduce the recall bias associated with the study measures, subjects were required to complete the patient diary within 24 h of each attack as opposed to waiting to report their experiences until a study visit. This study was designed to mimic clinical use by allowing subjects to continue with their established prophylactic medication and to use the rescue medication of their choice. The mean results from all four attacks and at the follow-up visit were similar, which suggests that the results are valid.

A limitation in this study was that the global satisfaction question asked at enrolment and the follow-up global question were worded slightly differently. The PPMQ-R includes a global satisfaction question that asks subjects to consider their overall satisfaction with the ‘study medication’. For screening, subjects were asked to consider their overall satisfaction with their current treatment regimen. The same 7-point scale was used for measuring subject responses.

Studying an early intervention paradigm presents a challenge because mild migraines may be self-limiting and other types of headache may be mistaken for mild migraines (19). However, a recent study of migraineurs found that 92% of headaches identified as migraines at early onset were confirmed migraines at headache peak (39). In our study, experienced migraineurs who reported a mild pain phase preceding moderate-to-severe migraines were recruited to limit the number of non-migraine headaches mistaken for migraines with mild pain at onset. However, by limiting the study to patients with migraine experience and a history of frequent and moderate or severe attacks, the study population included patients on the more severe end of the disease spectrum. The satisfaction and efficacy results were similar across four attacks and at study follow up. These findings suggest that the subjects in this study properly identified migraine headaches in the mild pain phase.

Obtaining information about the level of satisfaction with pharmaceutical products is reflective of the desire to consider the perspective of the patient in medical decision making. However, gathering information directly from patients presents methodological challenges. The act of asking questions about patients’ medication may impact their responses. To minimise the impact of examiner influence on the results of this study, we used a validated treatment satisfaction questionnaire.

Additional studies should be conducted to measure the relative importance of the impact of improvement in satisfaction related to dose, treatment paradigm or formulation. Although this study does not weigh the relative contribution of early intervention, appropriate dosing and rapid release formulation, the results suggest that patients who believe that sumatriptan was ineffective under different circumstances could be rechallenged under new treatment conditions before sumatriptan is declared suboptimal for the patient.

In conclusion, the results of this study suggest that for patients who are not completely satisfied with their current medication regimen, physicians have an opportunity to optimise a rapid and complete response by encouraging patients to treat migraine attacks using an early intervention paradigm and by prescribing the most effective dose and a faster dispersing, disintegrating and absorbing formulation.

## References

[1] Headache Classification Committee of the International Headache Society (2004). The International Classification of Headache Disorders. Cephalalgia.

[2] Stewart W, Lipton R, Celentano D, Reed M (1992). Prevalence of migraine headache in the United States. Relation to age, income, race, and other sociodemographic factors. JAMA.

[3] Stang P, Osterhaus J (1993). Impact of migraine in the United States: data from the National Health Interview Survey. Headache.

[4] Lipton R, Stewart W, Von Korff M (1997). Burden of migraine: societal costs and therapeutic opportunities. Neurology.

[5] Stang PE, Crown WH, Bizier R (2004). The family impact and costs of migraine. Am J Manag Care.

[6] Sheftell F, Bigal M, Tepper S, Rapoport A (2004). Sumatriptan: a decade of use and experience in the treatment of migraine. Expert Rev Neurother.

[7] (1991). Treatment of migraine attacks with sumatriptan. The Subcutaneous Sumatriptan International Study Group. N Engl J Med.

[8] Mathew NT, Dexter J, Couch J (1992). Dose ranging efficacy and safety of subcutaneous sumatriptan in the acute treatment of migraine. US Sumatriptan Research Group. Arch Neurol.

[9] Cady RK, Wendt JK, Kirchner JR (1991). Treatment of acute migraine with subcutaneous sumatriptan. JAMA.

[10] Ferrari M, Goadsby P, Roon K, Lipton R (2004). Triptans (serotonin, 5-HT1B/1D agonists) in migraine: detailed results and methods of a meta-analysis of 53 trials. Cephalalgia.

[11] Tfelt-Hansen P, Block G, Dahlöf C (2000). Guidelines for controlled trials of drugs in migraine: second edition. Cephalalgia.

[12] Cady R, Lipton R, Hall C (2000). Treatment of mild headache in disabled migraine sufferers: results of the Spectrum study. Headache.

[13] Martin V, Cady R, Mauskop A (2008). Efficacy of rizatriptan for menstrual migraine in an early intervention model: a prospective subgroup analysis of the rizatriptan TAME (treat a migraine early) studies. Headache.

[14] Dowson AJ, Mathew NT, Pascual J (2006). Review of clinical trials using early acute intervention with oral triptans for migraine management. Int J Clin Pract.

[15] Becker W, Christie S, Ahmad F (2003). Pain-free efficacy of sumatriptan in the treatment of migraine at the first sign of-pain: Prospective, double-blind, placebo-controlled Canadian multicenter study of sumatriptan 50 mg and 100 mg versus placebo (Abstract). Cephalalgia.

[16] Cady R, Sheftell F, Lipton R (2000). Effects of early intervention with sumatriptan on migraine pain: retrospective analyses of data from three clinical trials. Clin Ther.

[17] Scholpp J, Schellenberg R, Moeckesch B, Banik N (2004). Early treatment of a migraine attack while pain is still mild increases the efficacy of sumatriptan. Cephalalgia.

[18] Winner P, Landy S, Richardson M, Ames M (2005). Early intervention in migraine with sumatriptan tablets 50 mg versus 100 mg: a pooled analysis of data from six clinical trials. Clin Ther.

[19] Winner P, Mannix L, Putnam D (2003). Pain-free results with sumatriptan taken at the first sign of-migraine pain: 2 randomized, double-blind, placebo-controlled studies. Mayo Clin Proc.

[20] D’Amico D, Moschiano F, Usai S, Bussone G (2006). Treatment strategies in the acute therapy of migraine: stratified care and early intervention. Neurol Sci.

[21] Cady R, Sheftell F, Lipton R (2001). Economic implications of early treatment of migraine with sumatriptan tablets. Clin Ther.

[22] Halpern MT, Lipton RB, Cady RK (2002). Costs and outcomes of early versus delayed migraine treatment with sumatriptan. Headache.

[23] Landy S, Savani N, Shackelford S (2004). Efficacy and tolerability of sumatriptan tablets administered during the mild-pain phase of menstrually associated migraine. Int J Clin Pract.

[24] Kori S, McDonald S, Page R (2006). Gastric transit and absorption of reformulated and conventional sumatriptan tablets in migraineurs both during and outside of a migraine attack: evaluation by gastric scintigraphy (abstract?). Neurology.

[25] Barbanti P, Carpay AK, Kwong JW, Ahmad F, Boswell D (2004). Effects of a fast disintegrating/rapid release oral formulation of sumatriptan on functional ability in patients with migraine. Curr Med Res Opin.

[26] Carpay J, Schoenen J, Ahmad F (2004). Efficacy and tolerability of sumatriptan tablets in a fast-disintegrating, rapid-release formulation for the acute treatment of migraine: results of a multicenter, randomized, placebo-controlled study. Clin Ther.

[27] Shikiar R, Rentz A (2004). Satisfaction with medication: an overview of conceptual, methodological, and regulatory issues. Value Health.

[28] Food and Drug Administration (2006). Patient-Reported Outcome Measures: Use in Medical Product Development to Support Labeling Claims. Draft Guidance. Draft Guidance.

[29] Revicki DA, Kimel M, Beusterien K (2006). Validation of the Revised Patient Perception of Migraine Questionnaire: measuring satisfaction with acute migraine treatment. Headache.

[30] Revicki D, Kimel M, Chen W-H, McCormack J (2006). Validation of the Revised Patient Perception of Migraine Questionnaire for use in Clinical Trials: Measuring Satisfaction With Acute Migraine Treatment.

[31] MacGregor EA, Brandes J, Eikermann A (2003). Migraine prevalence and treatment patterns: the global Migraine and Zolmitriptan Evaluation survey. Headache.

[32] Pascual J, Munoz R, Leira R (2001). An open preference study with sumatriptan 50 mg and zolmitriptan 2.5 mg in 100 migraine patients. Cephalalgia.

[33] Pascual J, Bussone G, Hernandez J (2001). Rizatriptan-Sumatriptan Preference Study Group. Comparison of preference for rizatriptan 10-mg wafer versus sumatriptan 50-mg tablet in migraine. Eur Neurol.

[34] Luciani R, Osterhaus J, Gutterman D (1995). Patient preferences for migraine therapy: subcutaneous sumatriptan compared with other medications. J Fam Pract.

[35] Lipton R, Hamelsky S, Dayno J (2002). What do patients with migraine want from acute migraine treatment?. Headache.

[36] Davies G, Santanello N, Lipton R (2000). Determinants of patient satisfaction with migraine therapy. Cephalalgia.

[37] Hu X, Markson L, Lipton R (1999). Burden of migraine in the United States: disability and economic costs. Arch Intern Med.

[38] Kwong JW (2005). The effect of early intervention with sumatriptan tablets on migraine-associated productivity loss. J Occup Environ Med.

[39] Ng-Mak DS, Cady R, Chen Y-T (2007). Can migraineurs accurately identify their headaches as “migraine” at attack onset?. Headache.

